# Glutamine Synthetase Drugability beyond Its Active Site: Exploring Oligomerization Interfaces and Pockets

**DOI:** 10.3390/molecules21081028

**Published:** 2016-08-08

**Authors:** Cátia Moreira, Maria J. Ramos, Pedro A. Fernandes

**Affiliations:** UCIBIO, REQUIMTE, Departamento de Química e Bioquímica, Faculdade de Ciências, Universidade do Porto, 4169-007 Porto, Portugal; cds.moreira@fc.up.pt (C.M.); pafernan@fc.up.pt (P.A.F.)

**Keywords:** protein-protein interactions, computational alanine scanning mutagenesis, drugable pockets, glutamine synthetase, *Mycobacterium tuberculosis*, human, maize, inhibition, drug discovery

## Abstract

*Background*: Glutamine synthetase (GS) is a crucial enzyme to the nitrogen cycle with great commercial and pharmaceutical value. Current inhibitors target the active site, affecting GS activity indiscriminately in all organisms. As the active site is located at the interface between two monomers, the protein-protein interface (PPI) of GSs gains a new role, by providing new targets for enzyme inhibition. Exploring GSs PPI could allow for the development of inhibitors selective for specific organisms. Here we map the PPI of three GSs—human (hsGS), maize (zmGS) and *Mycobacterium tuberculosis* (mtGS)—and unravel new drugable pockets. *Methods*: The PPI binding free energy coming from key residues on three GSs from different organisms were mapped by computational alanine scan mutagenesis, applying a multiple dielectric constant MM-PBSA methodology. The most relevant residues for binding are referred as hot**-**spots. Drugable pockets on GS were detected with the Fpocket software. *Results and Conclusions*: A total of 23, 19 and 30 hot-spots were identified on hsGS, zmGS and mtGS PPI. Even possessing differences in the hot-spots, hsGS and zmGS PPI are overall very similar. On the other hand, mtGS PPI differs greatly from hsGS and zmGS PPI. A novel drugable pocket was detected on the mtGS PPI. It seems particularly promising for the development of selective anti-tuberculosis drugs given its location on a PPI region that is highly populated with hot-spots and is completely different from the hsGS and zmGS PPIs. Drugs targeting this pockets should be inactive on eukaryotic GS II enzymes.

## 1. Introduction

Glutamine Synthetase (GS, EC 6.3.1.2, also known as γ-glutamyl:ammonia ligase) is a widely spread metalloenzyme. Together with glutamate synthetase, GS plays a crucial role in the nitrogen and carbon cycle, actively controlling the levels of inorganic nitrogen incorporated into organic molecules by performing key reactions that influence both assimilation and ammonification processes. Various reactions are catalysed by GS. The most important and well-studied is the ATP-dependent synthesis of glutamine by combining glutamate and ammonium, commonly denominated as the biosynthetic reaction [1,2,3,4].

Different isoforms of this enzyme are expressed, varying in both sequence and structure. They are classified into three categories: GS I, with 450 to 470 residues per monomer, combined into homododecameric oligomers with 622 symmetry (two rings superimposed, each with 6 monomers); GS II, with 350 to 420 residues per monomer, arranged in homodecameric oligomers with 522 symmetry (two rings superimposed, each with five monomers); and GS III, containing around 700 residues per monomer, also organized in homodecameric oligomers (two rings superimposed, each with five monomers) but with the inter-ring interface head to head (instead of tail to tail, as found in GS I and GS II). Reports of nanotube-like supramolecular assemblies are also mentioned in the literature [5,6].

At first, it was believed that GS I was exclusive from prokaryotes and GS II was mostly found in eukaryotes, being GS III restricted to a few organisms from both kingdoms [7,8,9]. Today it is known that a single organism can encode more than one class of GS [9], and that the distinctive line which distributes GS classes in different kingdoms is more blurred.

The initial studies of GS were conducted only on GS I, more abundant in bacteria and easier to study, with the intent of gathering knowledge on the function of this rather important enzyme. Later on, GS studies began to bisect into two main areas of research: agriculture and medicine. In the agricultural field, studies aim to unravel GS II features, allowing for the improvement of crops yields, accomplished by using GS inhibitors as herbicides together with genetically modified crops resistant to the herbicide. Efforts have been pursued also to enhance the GS activity to improve crops yields by an increased assimilation of nitrogen (or to achieve the same yield with the use of smaller amounts of fertilizers). An example of this is the herbicide phosphinotricin, also known as glufosinate or BASTA^®^. Glufosinate, together with glyphosate, is one of the cornerstones of the billion dollar industry of genetically modified resistant to herbicides (GM-HR) crops [10]. Glufosinate is as large-spectrum herbicide used on weed control, which can be combined with GM-HR crops that tolerate this herbicide. However, since it can inhibit the GSs from any organism, their use to increase crop productivity is only confined to resistant crops. Additionally, there are major concerns about their safety. Evidences indicate that glufosinate may be a major danger with both environmental and human health repercussions [11,12,13,14,15,16]. In the pharmaceutical field, studies aimed to differentiate bacterial GS I from mammal GS II have been carried out with the expectation of developing new antibiotics, especially against tuberculosis where GS has proven, in vitro, to be an excellent target for anti-tuberculosis drug development [17,18,19,20,21]. Multiple efforts have been done in the last two decades to attain novel inhibitors of GS that can act as anti-tuberculosis therapeutic agents. A recent review on this topic, by Mowbray and co-workers [17], describes the inhibitors currently targeting mtGS. They can be divided into two categories, depending on the binding location. The first category encompasses amino acids analogues, like MSO or PPT, that act as suicide inhibitors that compete for the binding site of glutamate/glutamine, and once bound to GS become phosphorylated. Since this region of the active site is highly conserved amongst all GS, this inhibitors raise concerns about their safety. The second category is dedicated to inhibitors targeting the ATP binding site, which normally are bigger hydrophobic heterocycles that bind to a larger and less conserved region of the active site, aiming more selective inhibition than inhibitors such PPT or MSO. Inhibitors from both categories have been already co-crystalized with mtGS, providing insights about their action mechanism [21,22,23,24,25,26]. Either way, all of them bind in the active site or its vicinity. But caution is needed when developing drugs targeting GS inhibition, since irregularities in human GS activity/expression are associated with several diseases and its deficiency leads to neonatal death caused by multi-organ failure [27,28,29].

Since GSs possess such a variety of roles, so important in metabolism, they need to be subjected to tight regulation, acting on many levels, from the gene expression to enzymes modulation. There are insightful reviews on this matter for bacterial GS (mostly *E. coli* [5]) and plants GS [30,31]. In mammals, GS has distinct roles in the metabolism, depending on the tissue location: in the brain, it regulates the levels of toxic ammonia and neurotoxic glutamate, and in the liver it participates in the elimination of ammonia. There are several studies trying to create an integrated picture of human GS regulation, mostly directed to astrocytes and hepatic cells [32,33,34,35].

GS has stimulated scientific interest for many reasons—its crucial role, its complex regulation, its multiple structures and its potential to industry—leading several groups worldwide to dedicate their efforts to unravel more pieces of knowledge on GS, hoping that the combination of these pieces would allow the manipulation of GS activity to our benefit. Here, new pieces of the GS puzzle are supplied, providing information regarding the GS oligomerization interfaces and drugable pockets. Until today, all strategies for GS inhibition pass through targeting its active site. However, as it lies in a well-conserved region, between the C-terminus of one monomer and the N-terminus of the adjacent monomer, problems of selectivity towards distinct organisms became unavoidable. In order to achieve that kind of selectivity, less conserved regions need to be targeted for inhibition. Therefore, detailed knowledge about the PPI in three distinct GSs, from human, maize and tuberculosis pathogen, has been gathered here. The choice of these three GSs was made upon the availability of structural data, which is fundamental to accomplish this study, combined with the necessity to collect new information that could revolutionize the search for novel herbicides and anti-tuberculosis drugs. Additionally, novel pockets located outside the catalytic site were searched for and their characteristics were analysed in terms of drugability.

When analysing the PPI interactions, we need to look at the residues present in the interface and infer their importance to the establishment of that same interface. One of the best and most accepted ways to do so is by measuring the variation of the binding free energy of the complex induced by the mutation of a given residue to an alanine (ΔΔG_bind_), a residue with a small, almost non-interacting side chain. If a residue important for binding is mutated into an alanine, the binding free energy of the complex should rise, given that a stabilizing contribution is lost. That is the principle behind alanine scanning mutagenesis. Therefore, in order to properly evaluate the individual contribution of the residues found in hsGS, zmGS and mtGS PPI we need to: (1) identify the residues present in the interface; mutate them by alanine; (2) calculate the binding free energy for both the wild type and mutated complex; (3) compare the obtained binding free energies between the mutated complex and the wild type complex (ΔΔG_bind_). All analysed residues, from here onwards, will be classified as hot spots (HS)—if their mutation to alanine increases the binding free energy in 4 kcal·mol^−1^ or more—as warm spots (WS)—if their mutation to alanine results on an increase on the binding free energy between 2 and 4 kcal·mol^−1^—or as null spots (NS)—if their mutation to alanine does not increase the binding free energy in more than 2 kcal·mol^−1^. The intervals that define HS, WS and NS can vary from author to author, but the numbers chosen here are the most commonly used. It is commonly accepted that a variation superior to 2 kcal·mol^−1^ reveals important residues on the PPI [36,37,38,39]. A variation greater than 4.2 kcal·mol^−1^ will lower the association constant by at least 1000 fold.

The discovery of small-molecule inhibitors targeting PPI is a challenging goal to achieve. However, it is a strategy with increasing interest among computational chemists [38,40,41,42]. In fact, some recent works used computer simulations that allowed the discovery of cryptic drugable binding sites, that in some cases lead to FDA approved drugs [43,44,45,46]. Given the intrinsic importance of the PPI in GSs enzymes, plus the location of the active site across the PPI, development of small-molecule inhibitors targeting the less conserved GS PPI could allow the establishment of directed inhibitors that are specific for a subset of GSs. If this is achieved and GS oligomerization is inhibited or destabilized, disruption of GS activity is obtained by the non-formation or malformation of the active site. But to do that two main questions need to be answered: are the PPI on GS belonging to distinct organism different enough? Which are the distinctive features of different GSs enzymes?

## 2. Results

Before analyzing the results some remarks have to be made regarding the residues nomenclature used in the following sections. Since we are studying PPI in a homo-oligomeric enzyme, interfacial interactions will be established between residues from equivalent monomers. A “central” monomer will be defined and colored in pink in all figures. The other binding partner will be named as “adjacent monomer”. If the adjacent interacting monomer is from the same ring (intra-ring interaction; monomer colored in blue in all figures), the residue from that monomer will be followed by a single quotation mark (e.g., Y100’). If the residue is arising from the adjacent interacting monomer located on the other ring (inter-ring interaction; monomer colored in green in all figures) an asterisk will be placed on the residue name (e.g., Y100*).

### 2.1. hsGS PPI

In hsGS PPI we found 54 distinct residues with potential to be relevant (buried area ≥ 40 Å^2^ [47]), taking into account all the 10 interacting monomers. 30 among these were common to at least 6 interfaces (Table 1). Taking into account the role of HS on the formation of stable PPIs, only these 30 residues were considered for the calculations. Note that the number of residues here discussed do not include glycine, proline and alanine since their impact on the energy binding is not possible to be measured by computational alanine scanning mutagenesis (cASM). The Ala/Ala mutation is irrelevant, the Gly/Ala mutation will in fact increase the side chain, and rigidify the backbone, which might result in changes in the folding, and the Pro/Ala will most probably change the backbone folding as well. Two distinct interfaces exist in an hsGS dodecamer: intra-ring interface and inter-ring interface.

The inter-ring interface of hsGS is established by the loop L139-P160 that has a high content on both glycine and proline residues. From this interface, the contribution of T142, N151 and F153 to the binding free energy was calculated, revealing only small contributions (Table 1). No other residues, aside from the G and P, appear to stand out as important in the role of stabilizing hsGS inter-ring interfaces. This interface should result from a combination of multiple small contributions from this loop residues. This fact is supported by the literature that states that both proline and glycine are residues commonly found in PPI, being able to stabilize protein-protein interactions [48,49,50], most probably through a hydrophobic effect.

**Figure 1:**
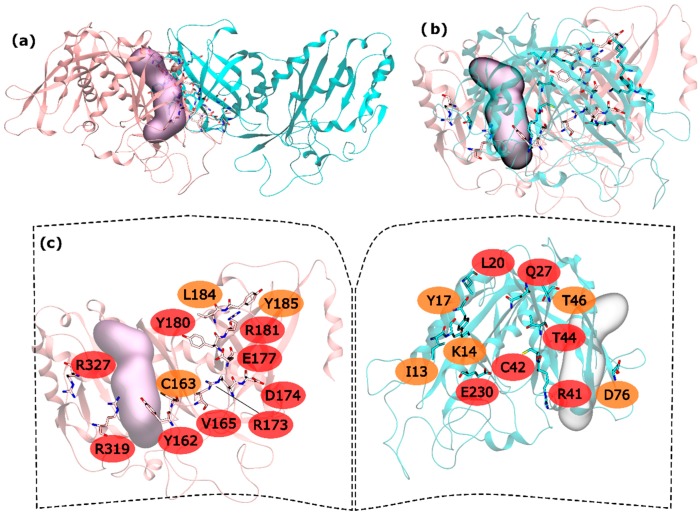
HS and WS detected on the hsGS intra-ring PPI. Null spots were omitted. (**a**) front view of the hsGS intra-ring PPI of adjacent monomers; (**b**) side view of the hsGS intra-ring PPI of adjacent monomers; and (**c**) “open book” view of the hsGS intra-ring PPI with the identification of the HS (red tag names) and WS (orange tag names); The active center location is given by the purple surface (defined by ADP, the methionine sulfoximine inhibitor and three Mg^2+^ cations). Distinct monomers are colored in pink and cyan.

Regarding hsGS intra-ring interfaces, they are established by adjacent monomers on the pentameric ring, in which a C-terminus of a monomer interacts with the N-terminus of the adjacent right monomer (Figure 1). From the 30 residues with potential to be HS, 15 are very important to the interaction in PPI (and classified as HS) and 8 seem to be important, albeit in a smaller extent than the hot spots (classified as WS). In the N-terminus side, L20, Q27, R41, C42, T44 and E230 were the detected HS and I13, K14, Y17, T46 and D76 ranked as WS (Figure 1). In the C-terminus, Y162, V165, R173, D174, E177, Y180, R181, R319 and R327 were found to be HS and C163, L184 and Y185 to be WS (Figure 1).

The majority of the HS and WS are located in the inner part of the intra-ring interface PPI, closer to the center of the ring, having only R327 and R319 interacting with D76′ of the adjacent monomer in the outer part of the PPI. On this interaction, D76′ side chain carboxylic group interacts with the side chain amide group of the R319 and the backbone N from R327. The side chain amide group of R327 also interacts with the backbone oxygen of D76′. Therefore, a number of strong interactions are observed between these three residues. The outer part of the PPI is separated from the inner part by the active site. In the inner part of the PPI, a complex network of interactions is established between the resident HS and WS from both monomers. Moving from the active site to the center of the ring structure, interactions are observed between Y162-R41′, C163-C42′, Y180-T46′, E177-Q27′-R181-L20′-Y185, R173-E230′ and D174-K14′. Hydrogen bonds are seen between: R41′ terminal amide group and Y162 backbone oxygen; backbone oxygen of C163 and backbone amide from C42′; side chain Y180 and backbone amine of T46; E177 side chain carboxylic group with Q27′ side chain amide group; Q27′ side chain carbonyl group and terminal amide of R181 that also interacts with backbone carboxyl group of L20; and finally, K14′ side chain amide with side chain carboxylic group of D174. C163-C42’ interactions has already been described as important on other studies performed on a distinct organism, reveling that disulfide bridges between this two residues can regulate GS activity [51]. Some aliphatic-π interactions are also observed between L20′-Y185 side chains. These are only the interactions established between HS and WS residues of distinct monomers. Some WS contribute to the PPI by interacting with HS of the same monomer, stabilizing it. This is exemplified by these two examples in which I13′ and L184 are interacting with Y17′ and Y180, respectively. From these results, it is possible to see that the intra-ring interface is the desirable place to develop PPI inhibitors to inhibit hsGS that would not only inhibit the oligomerization but also disrupt the activity of this enzyme (given that the catalytic site is located in this interface).

In zmGS, 67 distinct residues were found on the PPI that have potential to be relevant, taking into account all 10 interacting monomers. However, only 34 residues among these have the potential to be considered HS (Table 2). Similarly to what happens in hsGS, neither HS nor WS were found in the inter-ring interface, due to the intrinsic limitations of the ASM methodology. Once again, this interface is composed solely by the interaction of one loop (L132-P156) that is rich in both G and P residues. I147 and F150 were the only residues tested, falling under the NS classification.

### 2.2. zmGS PPI

**Figure 2:**
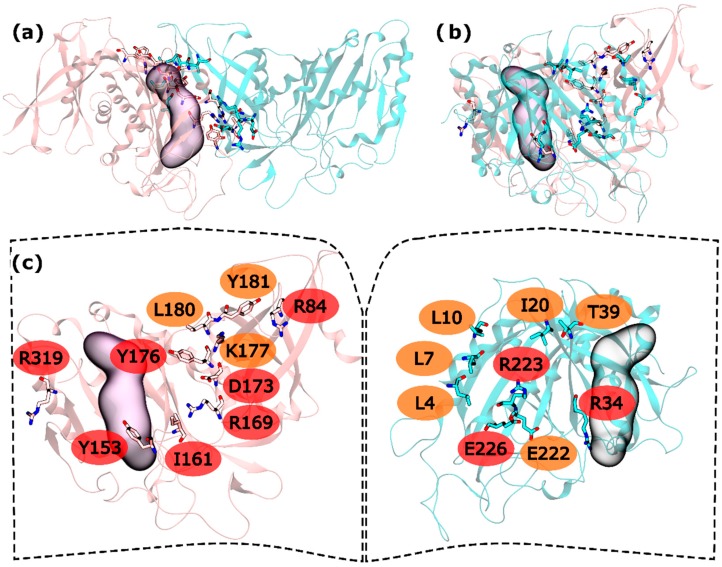
HS and WS detected on the zmGS intra-ring PPI. Null spots were omitted. (**a**) front view of the hsGS intra-ring PPI of adjacent monomers; (**b**) side view of the hsGS intra-ring PPI of adjacent monomers; and (**c**) “open book” view of the hsGS intra-ring PPI with the identification of the HS (red tag names) and WS (orange tag names); The active center location is given by the purple surface (defined by ADP, the methionine sulfoximine inhibitor and three Mg^2+^ cations). Distinct monomers are colored in pink and cyan.

In the intra-ring interface 32 residues were tested. From these residues, L4, L7, L10, I20, T39, and E222 were found to be WS on the N-terminus and K177, L180 and Y181 as C-terminus WS. HS were also found in both terminus: R34, R223 and E226 in the N-terminus and R84, Y158, I161, R169, D173, Y176 and R319 in the C-terminus (Figure 2). When compared with the hsGS intra-ring PPI, zmGS intra-ring PPI is at the same time similar but poor in both interactions and HS residues. Again, it is possible to see two distinct parts of the interface separated by the active site, one smaller outer part, away from the ring centre, and a bigger inner part, closer to the ring centre. In the outer part of the zmGS interface, only R319 was detected as HS. R319 interacts with D67 side chain by a hydrogen bond. On the inner part of the zmGS PPI, some of the observed interactions were the same as in hsGS PPI. It is the case of Y158-R34′ (equivalent to Y162-R41′ on hsGS), Y176-T39′ (Y180-T46′ on hsGS), R169-E226′ (R173-E230′ on hsGS) and Y181-L10′ (Y185-L20′ on hsGS). Nevertheless, distinct HS and WS were detected and with them distinct interactions were made in the zmGS intra-ring PPI. Such is the case of I20′ that established aliphatic-π interactions with Y176. Furthermore, the R223′ side chain interacted with the carboxyl group of D177. Another distinct interaction was that between the L10′ backbone and K177 side-chain. Also, notice that the previous interacting pair of cysteines found on hsGS was not detected on zmGS. Cysteine residues are described as important regarding GS regulation and their content can vary largely amongst GSs (check file S2 in the Supplementary Material).

Again, PPI inhibitors targeting the inter-ring interface of zmGS do not seem to be successful (as in hsGS): the high glycine and proline content plus the conservation level between the human and the maize enzyme makes it impossible to rationally design inhibitors targeting this interface. Inhibitors targeting this interface could be developed if distinctive features between hsGS and zmGS associated with drugable pockets could be found. However, such circumstances were not met (see below).

### 2.3. mtGS PPI

Finally, 42 residues were studied by cASM in the mtGS oligomer (Table 3). From that, a total of 19 HS and 11 WS were detected per monomer (475 residues). For the first and only time in this study, both HS and WS could be detected in the inter-ring interface. In fact, it is difficult to fully separate inter-ring from intra-ring interface, since there is a portion of HS/WS detected on the intersection of three interacting monomers, two of them belonging to the same hexameric ring. Given that it is simpler to draw a line separating the region containing only intra-ring PPI interactions, the region that resulted from the intersection of three monomers will be included on the inter-ring PPI results.

**Figure 3:**
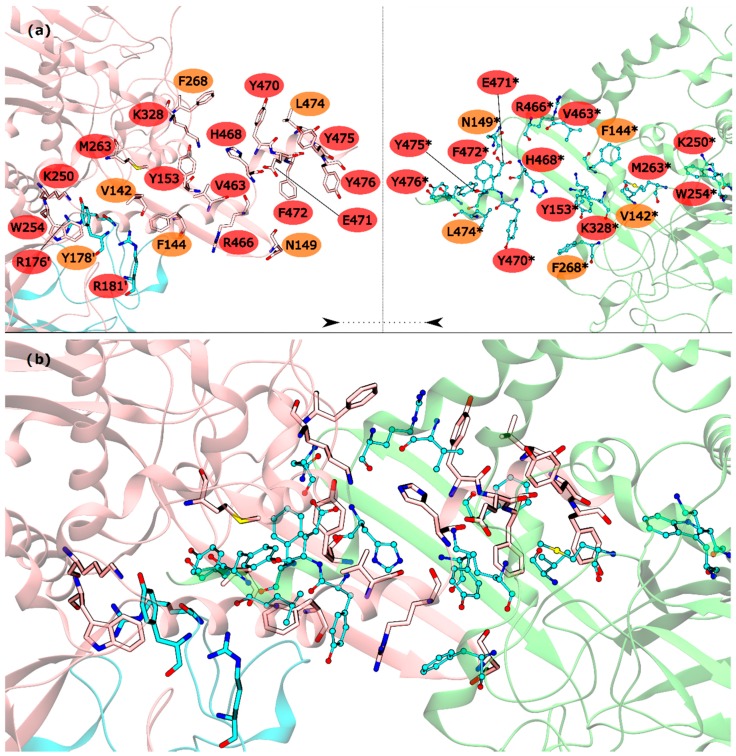
HS and WS detected on the mtGS inter-ring PPI. Null spots were omitted. (**a**) identification of the HS (red) and WS (orange) residues on this interface with the monomers separated by rings (pink and blue monomers belongs to the upper ring and green monomer to the lower ring); (**b**) representation of the mtGS inter-ring PPI. The HS and WS from the upper ring were represented in licorice, with the carbons colored by chain. The HS and WS from the lower ring are represented as ball and stick.

The HS detected in the inter-ring interface were Y153, R176′, R181′, K250, W254, M263, K328, V463, R466, H468, Y470, E471, F472, Y475 and Y476. The list of WS detected in the inter-ring interface was less extent, with only V142, F144, N149, Y178′, F268 and L474 (Figure 3). From these residues, V142, R176′, Y178′, R181′, K250, W254, L474 and Y476 are located on the intersection between inter-ring and intra-ring PPI. In this part of the mtGS PPI the most eye catching interactions are: cation-π interactions established between R176′-W254, a chain of hydrogen bonds involving Y178′-K250-D477*-R181′. D477 was not classified as HS or WS probably because its mutation D477A changed its hydrogen bond interaction with R181′ (between both side chains) into an aliphatic-π interaction of alanine with Y178′. Let’s move on to the pure inter-ring PPI, where only interactions are made between two monomers from distinct hexametric rings. The pure inter-ring PPI in mtGS is very distinct from the previous characterized eukaryotic ones. Just by looking at the 3-D structure of mtGS and the eukaryote GS’s it is possible to see that the intra-ring surface is much more extensive in mtGS. Combined analysis on both residues sequence and the 3-D structure of the monomer shows that mtGS possesses two extra interaction regions to establish inter-ring interactions (file S3 on support information). One is an expansion with 25 additional residues on the inter-ring interacting loop found in both hsGS and zmGS into a β-hairpin structure that aligns with the equivalent β-hairpin structure of the other ring’s adjacent subunit, forming together a β-meander motif in the inner part of the double ring oligomer and allowing more stabilizing contacts between the two rings. This β-hairpin structure contributes only with one HS (Y153) and three WS (V142, F144 and N149). The HS Y153 is interacting with F472* by π-π stacking of the phenol and benzyl rings. N149 is located at the hairpin loop and positioned towards interacting with the other subunit (by establishing aliphatic-π interactions with W162* and/or hydrogen bonds with D156*). V142 and F144 are located at the beginning of this motif and both interact by aliphatic-π interactions with Y476* and F472*, respectively. Besides this β-hairpin structure, the inter-ring contact area is also established by an insertion of the C-terminal α-helix of mtGS that does not have an equivalent domain on eukaryotic GS’s. From these extra C-terminal residues, the last 16 residues penetrate the adjacent subunit of the other ring. On these C-terminal α-helix, residues V463, R466, H468, E471, F472, Y475 and Y476 are HS and Y470 and L474 are WS. The majority of interactions established on this interface, between residues belonging to different subunits, involve π interactions. This is the case for V463-H468* (aliphatic-π interactions), Y470-R466*-F268 (cation-π interactions), M263-F472*-Y153 (π stacking) and Y476-V142* (aliphatic-π interactions). Two hydrogen bonds are also established between the two monomers by interaction of the side chains of residues E471-R328* and backbone amide R466-H468* imidazole side chain. From this C-terminus it remains uncharacterized the influence of terminal V478. This residue established an inter-monomer hydrogen bond with K250*, between the V478 backbone carboxylic end group and the side-chain amide of K250*, so performing the mutation V478A would not correctly measure this residue influence. Besides the residues from the β-hairpin and C-terminus, four other residues are important in this inter-ring PPI, belonging to different portion of the monomer. This is the case of K250, M263 and K328, which are all classified as HS, and F268 that is classified as a WS.

**Figure 4:**
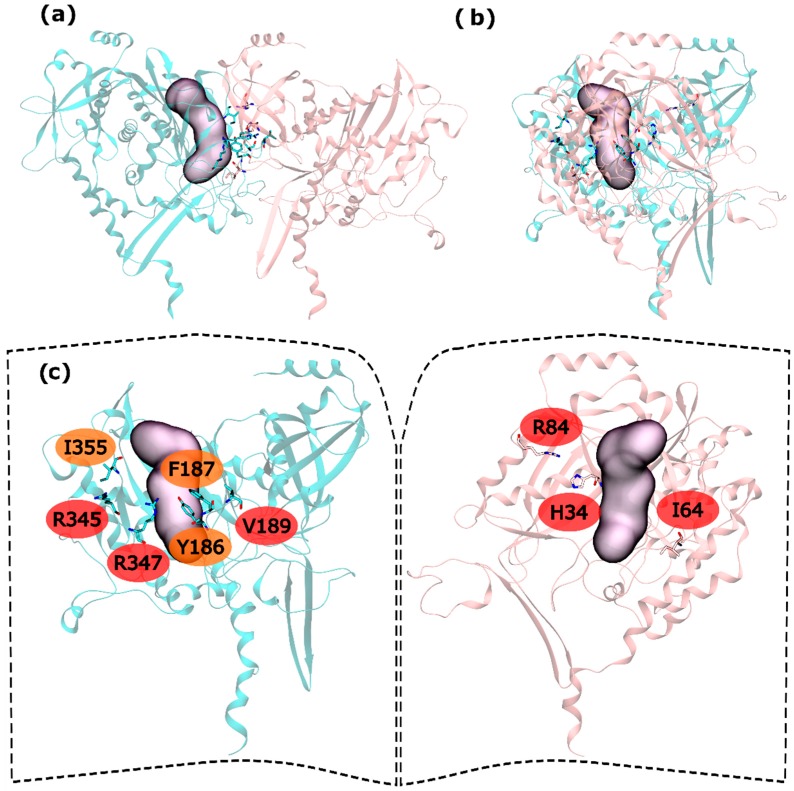
HS and WS detected on the mtGS intra-ring PPI. Null spots were omitted. (**a**) front view of the hsGS intra-ring PPI of adjacent monomers; (**b**) side view of the hsGS intra-ring PPI of adjacent monomers; and (**c**) “open book” view of the hsGS intra-ring PPI with the identification of the HS (red tag names) and WS (orange tag names); The active center location is given by the purple surface (defined by ADP, the methionine sulfoximine inhibitor and three Mg^2+^ cations). Distinct monomers are colored in pink and cyan.

Hot and warm residues were found also on the intra-ring interface search. In the N-terminus part, residues H34, I64 and R84 were detected as HS. In the C-terminus part V189, R345 and R347 were classified as HS and Y186, F187 and I355 fall into the WS classification (Figure 4). This gives six HS and three WS per intra-ring interface, a rather smaller content of both HS and WS compared with both hsGS (thirteen HS and eight WS) and zmGS (nine HS and nine WS). Hydrogen bond interactions are established between R345′-D68-R347′-I64 and R84-D200′. Residue I355′ establishes aliphatic-π interactions with D200.

In the case of mtGS, major differences were identified on inter-ring PPI that are worthy of being further explored as potential targets for PPI inhibitors. The intra-ring mtGS PPI is also distinct from the previous studied eukaryotic GSs, being poorer on both HS and WS. All this information of PPI from these three GS is of great value and could change the tide on drug discovery against GS enzymes.

### 2.4. Novel Drugable Pockets

To gather useful information that allows for the development of novel GS inhibitors with selectivity towards the GS from a single organism or at least a small group of organisms, novel drugable pockets, away from the active site, should be scanned. To achieve that goal, the Fpocket software was employed using an isolated monomer as target from all three studied GSs (hsGS, zmGS and mtGS). Pockets were detected in all GSs, with a maximum number of pocket of 11 on hsGS (chain C), 12 pockets on zmGS (chain A) and 24 pockets on mtGS (chain F). From the detected pockets, only those with a drugability score above 0.5 were considered as drugable pockets [52].

**Figure 5:**
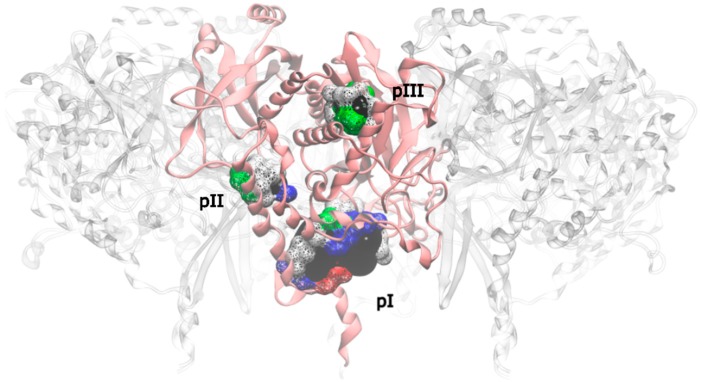
Drugable pockets or cavities identified on the mtGS monomer; the pocket volume is represented by a black surface; the residues that delimitate the pocket are represented in wireframe surface and colored by its nature (red if it is acidic, blue if it is basic, green if it is polar and white if it is nonpolar).

Applying this restriction, one drugable pocket was detected on hsGS (drugability score around 0.605 ± 0.001) and another one on zmGS (drugability score around 0.622 ± 0.032). In both cases, the location of the drugable pocket was overlapping the active site position and so they do not really constitute new pockets in terms of a new inhibition strategy. On mtGS, three drugable pockets were detected, which will be named pI, pII and pIII (Figure 5). None of this pocket has coincided with the active site location.

From those three drugable pockets, pI has a drugability score of 0.720 ± 0.100, being the larger drugable pocket found on the mtGS monomer. The residues that constitute this pocket are V142-V155, M263, K265-F268, N325, K328, P462, V463-Y470 and F472. The total SASA of pI is 750.64 Å^2^ (188.91 Å^2^ is polar SASA and 561.73 Å^2^ is apolar SASA), the volume is 1276.20 Å^3^. In order to investigate the molecular properties that good inhibitors should have, a virtual screening was carried out, using three libraries of molecules designed to cover a wide range of drug-like properties (ZDD, ZMD and NCI diversity 3 (ncidiv) subsets from ZINC database). All the ligands that have successfully bound the three mtGS pockets are given in S.I., as well as the measured affinity and a set of relevant ligand descriptors: molecular weight (MW, in Dalton), partition coefficient (LogP, measures the relative solubility in octanol/water), the apolar desolvation energy (desolv_apolar, in kcal/mol), the polar desolvation energy (desolv_polar, in kcal/mol), the number of hydrogen bond donors (HBD) and hydrogen bond acceptors (HBA), the topological polar surface area (tPSA, in Å^2^), the net charge of the molecules and the number of rotatable bonds (NRB).

All ligands were able to bind the mtGS pI pocket with reasonable to good affinity. The capability of the ligand to bind the pockets was indicated by the affinity calculated by AutoDock Vina, which corresponds to the (approximated) binding free energy between the receptor and the ligand. The results are given in S.I. There was a clear correlations between molecular weight and affinity, with the larger ligands (around 500 Dalton) on the top of the affinity list. This is consistent with the large size of pI.

A similar trend was observed when looking at the water-octanol partition coefficient (logP), a surrogate of the hydrophobicity of the ligand. Ligands with positive logP have high affinity to the pocket pI, with the best inhibitors showing logP values of 2. There was not a clear correlation between the number of hydrogen bond donors (HBD) and acceptors (HBA), and affinity. However, we can see that the whole pocket presents 7 HBD and 7 HBA.

The polar surface area (PSA) was also tested but does not seem to influence the binding affinity to this pocket.

Pocket pII was only found on chain E, with a drugability score of 0.614 and being composed by residues L28, P29, G60, T450, W451, F454 and K455. With a volume of 106.17 Å^3^, this is substantially smaller than pI pocket, having a total SASA of 16.91 Å^2^ only of apolar nature. The range of molecular properties that are ideal for inhibitors targeting pII were also investigated by virtual screening, with the same compound libraries. In this pocket not all the ligands tested were able to bind. The percentage of ligands that were able to bind mtGS pII was: 38% of zdd library, 65% of zmd library and 44% of ncidiv library. The preferred molecular weight is around 200 Dalton, but can vary from 50 to 570 Dalton. Also a logP near 0 was found to be more suitable for molecules that can be ligands to pII.

At last, pIII presented a drugability score of 0.636 ± 0.076, located in a region delimited by residues A128-F130, N279-L281, M289, T301, A302, Y305, L365, L384 and I391. This pocket is substantially smaller than pI and with similar dimensions of pII, having only a volume of 93.09 Å^3^ with a total SASA of 16.34 Å^2^ (4.57 Å^2^ is polar SASA and 11.77 Å^2^ is apolar SASA). The ligands that bind this pocket are scarce, in particular when compared to the previous pockets. Only a small percentage of the ligands was able to bind pIII (1.2% of the zdd library, 5.7% of the zmd library and 0.1% of the ncidiv library). The molecular weight can go from 50 to 250 Dalton, with a preference for smaller molecules. The range of logP for binders is wide, it goes from −4 to 3, with a preference towards hydrophilic molecules.

Both pII and pIII are small pockets, with a volume unfavorable to fit a larger sized drug. They are able to bind small sized drugs, but only with reasonable to low affinity. Even having similar volume, pII pocket fits larger molecules than pI, given that its location is nearer to the surface that pIII. Drugs to this two pockets would be difficult to achieve. On the other hand, pI looks very promising. The volume of pI allows fitting a molecule that could reach a molecular weight of 1000 Dalton, which is about two times the size of a large bioavailable drug. Furthermore, the polar SASA of pI is similar, but above the 140 Å^2^ threshold for bioavailability [53], which means that the pocket can accommodate a large bioavailable drug and has enough hydrogen bond donors and acceptors to satisfy typical druglike compounds.

## 3. Discussion

As mentioned before, PPI interactions were studied on three distinct GSs from three distinct organisms: human, maize and the bacteria responsible for tuberculosis. All three enzymes depend on oligomerization to become active, given that the active site is located in between monomers. Inhibitors directed to this enzyme are currently on high demand, as can be seen by the number of recent papers dedicated to design new inhibitors of GS [14,17,18,20,21,22,23,24,26,54,55,56,57,58,59]. However, all of them are designed to act on the GS active site, lacking on specificity against one particular organism or group of organisms. Attempts have been made to develop specific mtGS inhibitors by exploiting the small differences in the active site residues located in the ATP/ADP binding region. However, given the level of conservation seen on the active site, development of such specific inhibitors seems quite challenging.

Another approach would be targeting the PPI with small inhibitors directed to interact specifically with HS, blocking the oligomerization and subsequently the active site formation. This is the focus of this work.

From the results presented here it is clear that significant differences exist between prokaryotic GSI and eukaryotic GSII PPIs, mostly at inter-ring PPI. This PPI in GSII is almost inexistent, consisting only of a contact between two loops rich in glycine and proline. Glycine rich linkers are already been documented and are common in proteins [60]. Their function relies mainly on connecting distinct domains inside a protein but still allowing them to be flexible [60]. In both zmGS and hsGS the inter-ring interacting loop constitutes a linker between the C-terminus and N-terminus domain (file S2 on support information), a probable reason to have such a high amount of glycine in its constitution. The second most common residue in this loop is proline. Contrary to glycine, proline-rich motifs are described as critical in PPI associated with intracellular signaling and pathways [48,49,50]. It is possible that proline is the main responsible for the establishment of inter-ring PPI on eukaryotic GSs. Naturally disordered motifs have been described as important on PPI associated with signaling [61,62,63,64,65,66]. Despite their great interest, their disordered nature makes it difficult, if not impossible, to rationally design inhibitors that target them.

On the other hand, the inter-ring PPI of mtGS looks very promising as a potential target for the development of oligomerization inhibitors. A vast inter-ring PPI was found on mtGS, full with HS and WS belonging to a β-hairpin (D140-A157), the C-terminus α-helix (V463-V478), the end of the α-helix S234-W254, loop M263-F268 and a small α-helix (T323-V331). The first two (β-hairpin and C-terminus α-helix) are the motifs that are projected towards the other ring, being inserted on the other monomer. The remaining ones, together with the base of the β-hairpin and the C-terminus α-helix, form a “hat” that will receive the end part of the C-terminus α-helix from the other ring. From the drugable pockets detected here, the largest one is located on that “hat”. This pocket seems to be a very promising target for the binding of oligomerization inhibitors. More so, its inhibitors can be designed as small druglike molecules, or even as peptides or peptidomimetics. For instances, peptides based on the C-terminus α-helix could be explored as drugs. The fact that these peptides lie outside the Lipinski space is not a problem for the treatment of tuberculosis. As this is a lung infection, it is perfectly viable to perform the administration through the upper respiratory tract. 

Even if an inhibitor is successfully designed to act in this pocket, one can always hypothesize that it will only eliminate the double ring formation, keeping the active site assembled. This would not be an ideal situation, but it is important to keep in mind that: (1) the existence of such a big and important interface must have an influence on the activity of mtGS; (2) the inter-ring interface is close to the intra-ring interface, having a region in which contacts between 3 distinct monomers do exist; and (3) any destabilization induced on the mtGS tertiary and quaternary structures should propagate to the intra-ring dimerization (i.e., to the active site) and alter the catalytic activity. It is also important to notice that the mtGS studied here is encoded by glnA1 gene. There are studies point out glnA1 as the only essential GS in *Mycobacterium tuberculosis* [67]. So inhibitors targeting the mtGS model here studied should be enough to tackle the tuberculosis problem. An alignment of the four existent mtGS can be found on file S3 on support information.

The intra-ring PPI is much more conserved along GSs from different organisms. Still, some differences can be seen on both the HS/WS detection and on the comparative analysis of the three sequences (file S2 on support information). The N-terminus seems by far the most distinct portion of this PPI on the three GSs. The only coinciding HS/WS found were R41 and T46 of hsGS with R34 and T39 of zmGS. The C-terminus portion of the intra-ring interaction, on other hand, has a more conserved sequence and the presence of HS/WS, being almost identical on the two eukaryotic GS’s studied. MtGS lacks a whole region of HS/WS (R173-Y185) on hsGS nomenclature. But, given that no drugable pockets were detected here, inhibition of mtGS hexameric ring oligomerization seems difficult to be achieved. Nevertheless, intra-ring PPI seems to be stronger in hsGS and zmGS than in mtGS, so any destabilization in this bacterial enzyme quaternary structure could be of great importance. Either way, this novel knowledge can definitely guide future investigations regarding the development of oligomerization inhibitors towards GS enzymes.

Some remarks have to be made regarding the transferability of this new pieces of the GS puzzle to other enzymes that were not included on this study. An alignment containing sequences of GS belonging to distinct organism that have at least one x-ray crystallographic structure deposited on the PDB was made and is available on file S3 on support information.

## 4. Materials and Methods

### 4.1. Selection of Crystallographic Structures

**Figure 6:**
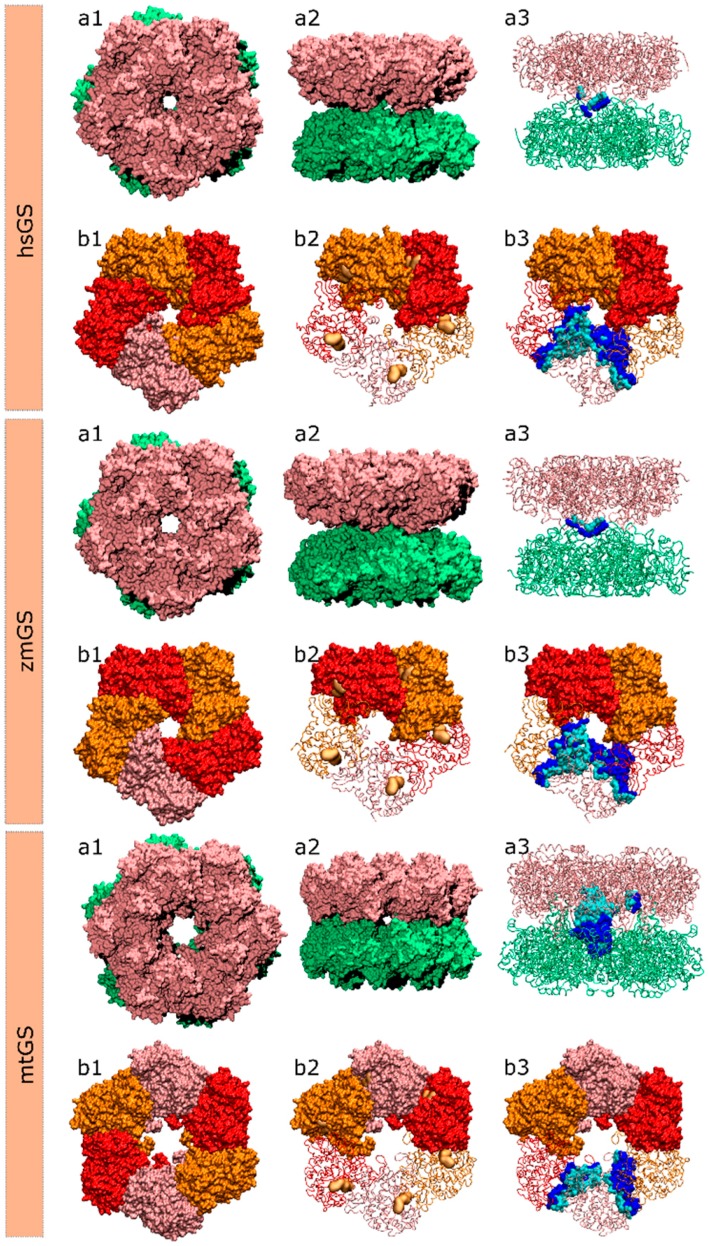
Structure of the hsGS, zmGS and mtGS double ring (**a**) or single ring (**b**) oligomers showing: (**a1**) top view; (**a2**) side view; (**a3**) highlight of the inter-ring contact surface between one monomer A of the above ring and the lower ring monomers, defined by all residues of the monomer A (cyan)/lower ring monomers (blue) that are within 5 Å distance from each other; (**b1**) ring top view with distinct monomers highlighted by colour; (**b2**) the active site location in between two adjacent monomers is demonstrated by a yellow surface delimiting the binding area of glutamate, ATP and metal ions; and (**b3**) highlight of the intra-ring contact surface between monomer A and the adjacent monomers of the same ring, with the residues of monomer A within 5 Å distance from the adjacent monomers coloured in cyan and the residues, also within 5 Å, of the adjacent monomers to monomer A coloured in blue.

Different GS crystallographic structures were chosen from the Protein Data Bank website, one prokaryotic GS I from *Mycobacterium tuberculosis* (mtGS; PDB accession code 2BVC, 2.1 Å resolution [25]) and two eukaryotic GS II from *Zea mays* (zmGS; PDB accession code 2D3A, 2.63 Å resolution [68]) and *Homo Sapiens* (hsGS; PDB accession code 2QC8, 2.6 Å resolution [69]). Figure 6 illustrates the structure of these three enzymes. All the three GS structures were crystallized with the methionine sulfoximine inhibitor, ADP and 2 or 3 divalent cations (Mg^2+^ or Mn^2+^) in the active site. The choice of these three species lays upon their potential interest by the industry that is searching both anti-tuberculosis agents and new herbicides. Also, it allows the study of both GSI (mtGS) and GSII (zmGS and hsGS).

### 4.2. Study of the Oligomerization Interface by cASM

**Figure A1:**
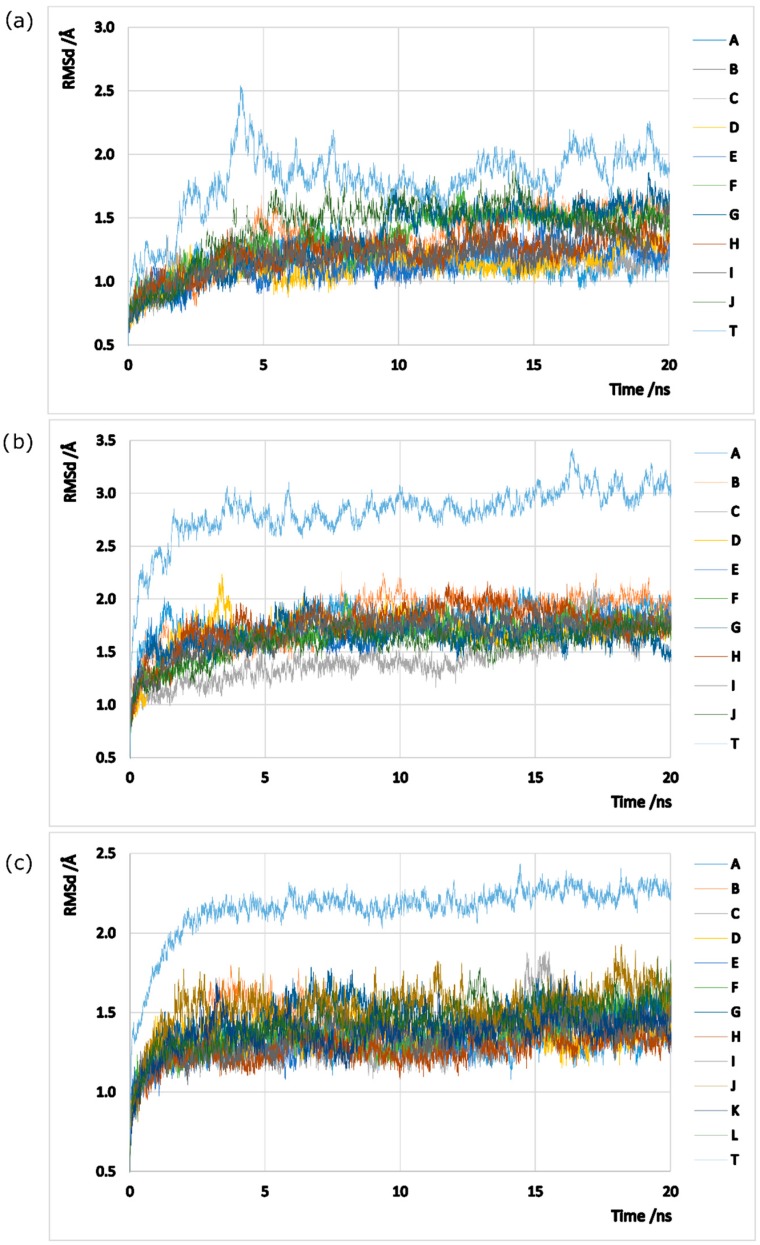
Graphic representation of the RMSd values along the time of MDs: (**a**) hsGS; (**b**) zmGS; and (**c**) mtGS. A-K represent the RMSd calculated to a single monomer and T represents the RMSd for the whole homo-oligomer.

Models were prepared based on the crystallized structures 2BVC, 2D3A and 2QC8 taken from the PDB website, solely composed by 10/12 protein chains arranged in a pentameric/hexameric double ring oligomer. All ligands and ions were stripped out from the models given that they are not fundamental for the GS oligomerization process. Note that GS enzymes can adopt distinct conformations, depending on their type/subtype and on the ligands that they are interacting with [3,69,70,71]. The no inclusion of any ligands or metals aims to study the native conformation of GS enzymes that is susceptive to monomer separation. Classical molecular dynamics simulations (MDs) were employed in all models in order to sample the phase space, using the AMBER 12 package [72]. Hydrogens (assuming physiological protonated states), counter ions and water type TIP3P molecules (a minimum distance of 12 Å was left between the protein and the water box edges, tallying X, Y and Z water molecules to the hsGS, zmGS and mtGS models) were added with the Leap program. The three GS enzymes were described by ff03.r1 [73] parameters. Minimizations on Sander were performed following a 4-stages minimization. Harmonic potentials (force constant of 50 kcal/mol/Å^2^) were used at first to restrain the positions of selected atoms in the systems, and later gradually removed: stage 1 (500 steps) all atoms except the ones from the water molecules were restrained; stage 2 (800 steps) the restrains at the hydrogen atoms were released; stage 3 (2500 steps) only the backbone chain atoms were restrained; and stage 4 (6000 steps) all the system was set free. After the system was fully optimized, 20 ps heating MDs, where temperature was raised from 0 to 300 K at constant volume, and 20 ns production MDs (NTP emsemble at 300K and 1bar controlled by Langevin thermostat and Berendsen barostat; 2 fs timestep, SHAKE algorithm used to fix the bond length between hydrogen atoms and heavy atoms) with PMEMD (Particle Mesh Ewald Molecular Dynamics) were performed on the models. For each model, 500 microstates were collected (every 8ps) in the initial (but with good equilibration in all chains) nanoseconds of MDs (between 12th and 16th ns on hsGS, between the 10.4th and 14.4th ns on zmGS and between 8th and 12th ns on mtGS). It was assured that a good sampling of statistically-independent microstates was carried out. The RMSd (calculated using cpptraj program) of the three models MDs, both for each individual chain and the whole system, can be consulted in Figure A1 in the Appendix.

The CompASM plugin [47] for VMD was employed to select the residues located on the interface. Each monomer was selected as ligand against the remaining subunits of the decamer/dodecamer (defined as receptor). Then the NCSA (non-solvent contact area) method was employed to select those residues among the ones located near the interface (within 5Å) that could strongly contribute to PPI establishment (i.e., the ones that bury at least 40 Å^2^ upon dimerization) [47]. The complete list of residues selected to each chain can be consulted in Table A1, Table A2 and Table A3 in the Appendix. This final selection of residues allowed us to shrink the list of residues tested, enriching the chosen ones in HS and WS. Since HS and WS are the important residues for dimerization, it is only natural that they should be found almost always in the interface.

Models for the complex, ligand and receptor were created to be submitted to cASM, containing only selected portions of the complete enzymes. In these models, the complexes contained 2 or 3 interacting chains (depending if we were mutating the intra-ring or inter-ring residues), the ligands model contained only one chain, and the receptor model contained 1 or 2 chains. The force field applied on MM-PBSA calculations was ff99SB [74]. 

Alanine mutations were performed on the selected residues, by manual deletion of all side-chain atoms of the target residue up to Cβ. The Cγ was replaced by a hydrogen. The MM-PBSA.py [60] script from the AMBER 12 package [58] was employed to calculate the ΔG_binding_ for each mutant and for the wild-type. Depending on the residue mutated, three internal dielectric constants were used (as suggested by Moreira et al. [26]): for charged residues—glutamine, aspartate, arginine, lysine and histidine—an internal dielectric 4 was employed; for polar residues—glutamine, asparagine, cysteine, tyrosine, serine and threonine—an internal dielectric 3 was used; and for apolar residues—valine, leucine, isoleucine, phenylalanine, methionine and tryptophan—an internal dielectric 2 was applied. For the solvent a dielectric constant of 80 was used. This method was shown to have comparable accuracy to much more demanding free-energy schemes such as Thermodynamic Integration, at a fraction of the computational cost [75], allowing it to be applied in the mutation of a very vast number of residues. ΔΔG_binding_ energies (Equation (1)), which allow to infer the contribution of the side-chain of residue to the establishment of the protein-protein association (Table 1, Table 2 and Table 3), were calculated as:

(1) $$\Delta \Delta G_{binding}=\Delta G_{mut}-\Delta G_{wt}$$

(2) $$\Delta G_{wt,mut}=G_{cpx}-\Delta G_{receptor}-\Delta G_{ligand}$$

(3) $$G=E_{internal}+E_{electrostatic}+E_{VDW}+G_{epolar\;solvation}+G_{nonpolar\;solvation}-TS$$

As the protein’s vibrational entropy is very difficult to calculate with satisfactory accuracy, we have not included it in the calculations. As such we have only calculated relative binding free energies, but not the absolute binding free energies. The basic assumption is that when one calculates the difference in binding free energy between the two association forms (wild type and mutated), the vibrational, rotational and translational entropy variations will mostly cancel out [36]. The solvation entropy is accounted for explicitly.

### 4.3. Search for Drugable Pockets

Crystallographic structures selected to integrate this study were analyzed by the Fpocket software [76] in order to explore new drugable sites. Fpocket is a very fast, easy and free tool that allows the identification of cavities on large proteins and protein complexes from a single PDB structure or from multiple structures sampled in MDs. It also allows the gathering of information on the cavity physico­chemical environment. A particularly useful descriptor provided by Fpocket, the drugability score [52], is of the utmost importance on this study. The search for pockets was made on a single monomer (repeated on the first six chains of each PDB). Each monomer was extracted from the X-ray structure of the oligomer, since the 3D structure of the monomer by itself was not determined so far.

### 4.4. Docking of Molecules on the Drugable Pockets

Three compound libraries, that are known to contain molecules with a vast range of physical/chemical properties, were selected as ligands from the ZINC Database: ZINC Drug Dataset (zdd), Zinc molecules dataset (zmd) and NCI Diversity set 3 (ncidiv). The receptor was a monomer extracted from the crystalized oligomer of mtGS. Openbabel [77] was employed on both ligands and receptor to add hydrogens at pH 7. Standard protonation states were used, with the ligands treated as flexible molecules and the receptor as a rigid molecule. The Autodock plugin to pymol was employed to select the region where the ligand docking should take place, region that is enclosure onto a box with the dimensions (in Å) of 26.25 × 18.75 × 18.75 centered on pI, 12.00 × 14.62 × 13.12 centered on pII and 13.50 × 12.00 × 13.50 centered on pIII. Molecular docking of the libraries of compounds into the three novel pockets of mtGS was performed employing AutoDock Vina [78]. Ligands were sorted by the affinity of their best pose and the ones that presented a measured affinity greater than 0 kcal/mol were excluded as putative ligands (since a positive affinity is an indicator that the ligand will not bind the receptor on that region). The molecular properties of ligands with a negative affinity to the pockets (that can be consulted on file S1 on support information) were then evaluated to obtain the range of properties that favor the binding of ligands to each mtGS new pockets. The analyzed properties are the molecular weight (MW, in Dalton), the partition coefficient (LogP, measures the relative solubility in octanol/water), the apolar desolvation energy (desolv_apolar, in kcal/mol), the polar desolvation energy (desolv_polar, in kcal/mol), the number of hydrogen bond donors (HBD) and hydrogen bond acceptors (HBA), the topological polar surface area (tPSA, in Å), the net charge of the molecule and the number of rotatable bonds (NRB).

## Figures and Tables

**Table 1:** Residues detected on hsGS PPI as a potential HS (buried area ≥ 40 Å^2^ in at least 6 monomers) with the respective mean value of ΔΔG_bind_ determined by cASM, standard error of mean (SEM) and their classification as HS (red), WS (orange) or NS (yellow).

Resid	ΔΔG_bind_	SEM	Classif
I13	3.82	0.40	WS
K14	2.67	0.29	WS
Y17	2.69	0.16	WS
M18	0.59	0.22	NS
L20	4.57	0.16	HS
Q22	1.38	0.04	NS
Q27	5.84	0.30	HS
R41	6.12	0.26	HS
C42	10.63	1.07	HS
K43	1.23	0.89	NS
T44	11.21	1.23	HS
T46	2.07	0.10	WS
S66	0.09	0.09	NS
D76	2.86	0.25	WS
N152	0.94	0.43	NS
F154	0.42	0.23	NS
Y162	4.37	0.18	HS
C163	2.22	0.11	WS
V165	5.29	0.15	HS
R173	4.22	0.32	HS
D174	5.15	0.41	HS
E177	9.52	0.53	HS
Y180	4.85	0.38	HS
R181	7.90	0.57	HS
L184	3.32	0.10	WS
Y185	3.54	0.07	WS
E230	12.47	0.58	HS
F232	−0.02	0.01	NS
R319	5.14	0.71	HS
R327	5.40	0.38	HS

**Table 2:** Residues detected on zmGS PPI as a potential HS (buried area ≥ 40 Å^2^ in at least 6 monomers) with the respective mean value of ΔΔG_bind_ determined by cASM, standard error of mean (SEM) and their classification as HS (red), WS (orange) or NS (yellow).

Resid	ΔΔG_bind_	SEM	Classif
C3	0.75	0.15	NS
L4	3.14	0.27	WS
D6	1.84	0.21	NS
L7	3.62	0.26	WS
V8	0.35	0.22	NS
L10	3.78	0.30	WS
L12	−4.30	0.45	NS
T15	−1.99	0.43	NS
I20	3.67	0.33	WS
R34	7.21	0.24	HS
T39	2.21	0.07	WS
D56	−0.10	0.50	NS
S59	0.59	0.19	NS
E69	2.42	0.78	NS
R84	4.74	0.23	HS
F150	1.26	0.29	NS
Y158	3.88	0.32	HS
C159	1.64	0.11	NS
I161	7.08	0.20	HS
E164	−1.68	0.44	NS
R169	8.28	0.25	HS
D170	−1.46	0.06	NS
D173	5.44	0.69	HS
Y176	4.07	0.20	HS
K177	3.39	0.40	WS
L180	3.91	0.23	WS
Y181	2.90	0.36	WS
E222	1.62	0.53	WS
R223	−4.57	0.90	NS
E226	6.27	0.53	HS
I227	0.57	0.21	NS
N310	−0.59	0.41	NS
R319	3.94	0.32	HS

**Table 3:** Residues detected on mtGS PPI as a potential HS (buried area ≥ 40 Å^2^ in at least 10 monomers) with the respective mean value of ΔΔG_bind_ determined by cASM, standard error of mean (SEM) and their classification as HS (red), WS (orange) or NS (yellow).

Resid	ΔΔG_bind_	SEM	Classif
Y20	0.41	0.20	NS
I31	1.15	0.32	NS
H34	4.12	0.31	HS
F35	1.88	0.13	NS
I64	4.48	0.40	HS
H65	1.20	0.30	NS
D68	−0.12	0.52	NS
R84	7.06	0.74	HS
F99	1.53	0.43	NS
V142	2.92	0.26	WS
F144	2.60	0.21	WS
N149	3.48	0.13	WS
S151	−0.09	0.07	NS
Y153	7.18	0.17	HS
E154	−0.33	0.27	NS
R176	6.96	1.20	HS
Y178	3.42	0.22	WS
R181	9.57	1.12	HS
Y186	3.79	0.26	WS
F187	3.84	0.29	WS
V189	3.71	0.21	WS
V196	0.85	0.24	NS
D200	−1.28	0.44	NS
K250	14.54	0.28	HS
W254	4.46	0.28	HS
M263	6.94	0.17	HS
F268	3.60	0.13	WS
K328	13.60	0.17	HS
R345	16.09	0.36	HS
R347	12.97	0.49	HS
I355	3.17	0.15	WS
V463	4.40	0.24	HS
I465	0.87	0.17	NS
R466	8.82	0.14	HS
H468	4.50	0.08	HS
Y470	3.89	0.06	WS
E471	9.67	0.29	HS
F472	5.54	0.09	HS
L474	2.06	0.14	WS
Y475	5.49	0.10	HS
Y476	5.74	0.29	HS
D477	−2.78	0.28	NS

## Supplementary Materials

Supplementary materials can be accessed at: www.mdpi.com/1420-3049/21/8/1028/s1.

## Appendix

**Table A1:** Residues detected as putative HS on hsGS PPI and their classification as HS, WS or NS. In the columns “A” to “J”, each one referring to a GS chain, an x indicates that the residue on that monomer was detected as a potential HS (buried area ≥ 40 Å^2^). If the residue was detected in at least 6 monomers, ΔΔG_bind_ was determined by cASM and his classification (HS/WS/NS) is presented in the last column.

Resid	A	B	C	D	E	F	G	H	I	J	Tested	Classif
10		x			x	x	x	x		x	no	
11	x				x	x	x		x	x	no	
13		x	x	x	x	x	x	x	x	x	yes	WS
14	x		x		x	x	x		x	x	yes	WS
15	x				x	x	x		x		no	
16		x		x		x	x		x	x	no	
17	x	x	x	x	x	x	x	x	x	x	yes	WS
18	x	x		x	x	x	x		x	x	yes	NS
20	x	x	x	x	x	x	x	x	x	x	yes	HS
22	x	x	x	x	x	x	x	x	x	x	yes	NS
25								x			no	
27		x	x	x	x	x	x	x	x	x	yes	HS
41	x	x	x	x	x	x	x	x	x	x	yes	HS
42		x	x	x	x	x	x	x	x	x	yes	HS
43		x	x	x		x	x	x	x	x	yes	NS
44		x	x	x	x	x		x	x	x	yes	HS
46	x	x	x	x	x	x	x	x	x	x	yes	WS
63					x	x	x			x	no	
66		x	x	x		x	x	x	x	x	yes	NS
73	x				x	x		x	x		no	
74				x							no	
76	x	x	x	x	x	x	x	x	x	x	yes	WS
78				x	x	x				x	no	
90				x			x		x	x	no	
91						x	x				no	
104		x									no	
142	x		x			x	x		x	x	no	
143					x	x					no	
145					x						no	
152	x	x	x	x		x	x	x	x	x	yes	NS
154	x	x	x			x	x	x	x	x	yes	NS
162	x	x	x	x	x	x	x	x	x	x	yes	HS
163	x	x	x	x	x	x	x	x	x	x	yes	WS
165	x	x	x	x	x	x	x	x	x	x	yes	HS
168		x	x					x		x	no	
173	x	x	x	x	x	x	x		x		yes	HS
174	x	x	x	x	x	x	x	x	x	x	yes	HS
177	x	x	x	x	x	x	x	x	x	x	yes	HS
180	x	x	x	x	x		x	x	x	x	yes	HS
181	x	x	x	x	x	x	x	x	x	x	yes	HS
184	x	x	x	x	x	x	x	x	x	x	yes	WS
185	x	x	x	x	x	x	x	x	x	x	yes	WS
193		x	x					x		x	no	
226			x	x					x	x	no	
227				x	x						no	
230	x	x	x	x	x	x	x	x	x	x	yes	HS
231		x	x		x	x				x	no	
232	x	x	x	x	x	x	x	x	x	x	yes	NS
235	x			x	x	x				x	no	
318								x			no	
319	x	x	x	x	x	x	x	x	x	x	yes	HS
324			x								no	
327	x	x	x	x	x	x	x	x	x	x	yes	HS
328								x			no	

**Table A2:** Residues detected as putative HS on zmGS PPI and their classification as HS, WS or NS. In the columns “A” to “J”, each one referring to a GS chain, an x indicates that the residue on that monomer was detected as a potential HS (buried area ≥ 40 Å^2^). If the residue was detected in at least 6 monomers, ΔΔG_bind_ was determined by cASM and his classification (HS/WS/NS) is presented in the last column.

Resid	A	B	C	D	E	F	G	H	I	J	Tested	Classif
3	x	x	x	x	x	x	x	x	x		yes	NS
4	x	x	x	x	x	x	x	x	x	x	yes	WS
5	x	x		x		x			x	x	no	
6	x	x	x	x	x	x	x	x			yes	NS
7	x	x	x	x	x	x	x	x	x	x	yes	
8	x	x	x	x	x	x	x	x	x	x	yes	NS
9						x					no	
10	x	x	x	x	x	x	x	x	x	x	yes	WS
12	x	x	x			x	x	x	x	x	yes	NS
15		x		x		x	x	x	x	x	yes	NS
16					x						no	
18							x	x	x	x	no	
20	x		x	x	x	x	x	x	x	x	yes	WS
32					x					x	no	
33					x					x	no	
34	x		x	x	x	x	x	x	x	x	yes	HS
35	x				x	x		x	x	x	no	
36				x	x		x	x	x	x	no	
38								x			no	
39	x	x	x	x	x	x	x	x	x	x	yes	WS
56	x	x	x	x	x		x	x	x		yes	NS
59	x	x	x	x	x	x	x	x	x	x	yes	NS
66			x		x	x	x		x		no	
67	x	x	x		x						no	
68		x									no	
69		x	x	x	x	x	x	x	x	x	yes	NS
79	x				x						no	
81								x			no	
82					x			x			no	
83			x	x	x	x	x			x	no	
84	x	x	x	x		x	x		x	x	yes	HS
137	x	x				x	x	x			no	
138		x	x			x	x			x	no	
139						x					no	
140						x			x		no	
141									x		no	
147	x			x	x	x	x		x		no	
148			x								no	
150		x		x	x	x	x	x	x	x	yes	NS
158	x	x	x	x	x	x	x	x	x	x	yes	HS
159	x	x	x	x	x			x	x	x	yes	NS
161	x	x	x	x	x	x	x	x	x	x	yes	HS
164	x	x	x	x	x	x	x	x	x	x	yes	NS
165	x		x	x	x			x	x		no	
169	x	x	x	x	x		x	x	x	x	yes	HS
170	x	x	x	x	x	x	x	x	x	x	yes	NS
173		x	x	x	x			x	x	x	yes	HS
176	x	x	x	x	x		x	x	x	x	yes	HS
177	x	x	x	x	x	x	x	x	x	x	yes	WS
180	x	x	x	x	x	x	x	x	x	x	yes	WS
181	x	x	x	x	x	x	x	x	x	x	yes	WS
187				x	x					x	no	
189	x		x	x	x			x		x	no	
190			x								no	
193	x										no	
201				x	x					x	no	
222		x	x	x	x		x	x	x	x	yes	WS
223			x		x	x	x	x	x	x	yes	NS
224					x						no	
226	x	x	x	x	x	x	x	x	x	x	yes	HS
227	x	x	x	x	x	x	x	x	x	x	yes	NS
229				x	x		x	x		x	no	
231	x		x				x	x			no	
310		x		x		x	x	x	x	x	yes	NS
311	x		x	x			x		x	x	no	
316					x						no	
319	x	x	x	x	x	x	x	x	x	x	yes	HS

**Table A3:** Residues detected as putative HS on mtGS PPI and their classification as HS, WS or NS. In the columns “A” to “L”, each one referring to a GS chain, an x indicates that the residue on that monomer was detected as a potential HS (buried area ≥ 40 Å^2^). If the residue was detected in at least 10 monomers, ΔΔG_bind_ was determined by cASM and his classification (HS/WS/NS) is presented in the last column.

Resid	A	B	C	D	E	F	G	H	I	J	K	L	Tested	Classif
20	x	x	x	x	x	x	x	x	x	x	x	x	Yes	NS
31	x	x	x	x	x	x	x	x	x	x	x	x	Yes	NS
32			x		x				x				No	
33					x		x						No	
34	x	x	x	x	x	x	x	x	x	x	x	x	Yes	HS
35	x		x	x	x	x	x	x	x	x	x	x	Yes	NS
36		x	x	x	x	x	x	x	x	x	x		No	
40					x		x				x		No	
54		x	x	x	x	x	x	x	x		x		No	
59					x	x	x	x	x				No	
64	x	x	x	x	x	x	x	x	x	x	x	x	Yes	HS
65	x	x	x	x	x	x	x	x	x	x	x	x	Yes	NS
66		x								x			No	
68	x	x	x	x	x	x	x	x	x	x	x	x	Yes	NS
84	x	x	x	x	x	x	x	x	x	x	x	x	Yes	HS
99	x	x	x	x	x	x	x	x	x	x	x	x	Yes	NS
141				x	x	x	x	x					No	
142	x	x		x	x	x	x	x	x	x	x	x	Yes	WS
143	x	x			x		x	x					No	
144	x	x	x	x	x	x	x	x	x	x	x	x	Yes	WS
146									x				No	
147	x		x	x		x	x			x			No	
149	x	x	x	x	x	x	x	x	x	x	x	x	Yes	WS
151	x	x	x	x	x		x	x	x	x	x	x	Yes	NS
152	x	x	x	x	x	x	x	x	x				No	
153	x	x	x	x	x	x	x	x	x	x	x	x	Yes	HS
154	x	x	x	x	x		x	x	x	x	x	x	Yes	NS
161	x		x	x	x		x		x		x		No	
162					x				x				No	
164	x	x	x	x	x	x	x		x	x		x	No	
175	x		x	x			x		x	x		x	No	
176	x	x	x	x	x	x	x	x	x	x	x	x	Yes	HS
178	x	x	x	x	x	x	x	x	x	x	x	x	Yes	WS
179	x				x	x	x				x		No	
181	x	x	x	x	x	x	x	x	x	x	x	x	Yes	HS
186	x	x	x	x	x	x	x	x	x	x	x	x	Yes	WS
187	x	x	x	x	x	x	x	x	x	x	x	x	Yes	WS
189	x	x	x	x	x	x	x	x	x	x	x	x	Yes	WS
194						x							No	
196	x	x	x	x	x	x	x	x	x	x		x	Yes	NS
197						x							No	
199				x				x			x	x	No	
200	x	x	x	x	x	x	x	x	x	x	x	x	Yes	NS
203	x	x	x	x	x	x	x			x		x	No	
207		x		x									No	
212				x					x				No	
213		x		x		x							No	
214	x			x	x	x						x	No	
215	x	x	x	x	x	x	x		x	x	x		No	
247					x		x	x	x	x			No	
250	x	x	x	x	x	x	x	x	x	x	x	x	Yes	HS
251	x		x	x			x		x		x		No	
254	x	x	x	x	x	x	x	x	x	x	x	x	Yes	HS
T261					x	x	x	x		x			No	
M263	x	x	x	x	x	x	x	x	x	x	x	x	Yes	HS
P265					x						x		No	
F268	x	x	x	x	x	x	x	x	x	x	x	x	Yes	WS
N325					x		x	x					No	
Y327								x					No	
K328	x	x	x	x	x	x	x		x	x	x	x	Yes	HS
V331			x		x				x				No	
R345	x	x	x	x	x	x	x	x	x	x	x	x	Yes	HS
N346			x										No	
R347	x	x	x	x	x	x	x	x	x	x	x	x	Yes	HS
R352	x			x									No	
I355	x	x	x	x	x	x	x	x	x	x	x	x	Yes	WS
T356				x									No	
D404		x		x	x	x		x		x	x	x	No	
T421	x		x		x						x		No	
Q422	x	x	x	x	x	x	x	x	x			x	No	
S424									x				No	
E457	x												No	
E459			x			x						x	No	
V463	x	x	x	x	x	x	x	x	x	x	x	x	Yes	HS
N464	x	x	x			x	x	x	x	x	x	x	No	
I465	x	x	x	x	x	x	x	x	x	x	x	x	Yes	NS
R466	x	x	x	x	x	x	x	x	x	x	x	x	Yes	HS
H468	x	x	x	x	x	x	x	x	x	x	x	x	Yes	HS
Y470	x	x	x	x	x	x	x	x	x	x	x	x	Yes	WS
E471	x	x	x	x	x	x	x	x	x	x	x	x	Yes	HS
F472	x	x	x	x	x	x	x	x	x	x	x	x	Yes	HS
L474	x	x		x	x	x	x	x	x	x	x	x	Yes	WS
Y475	x	x	x	x	x	x	x	x	x	x	x	x	Yes	HS
Y476	x	x	x	x	x	x	x	x	x	x	x	x	Yes	HS
D477	x	x	x	x	x	x	x	x	x	x	x	x	Yes	NS
V478	x	x	x	x	x	x	x	x	x	x	x	x	No	
